# Optical Study and Experimental Realization of Nanostructured Back Reflectors with Reduced Parasitic Losses for Silicon Thin Film Solar Cells

**DOI:** 10.3390/nano8080626

**Published:** 2018-08-18

**Authors:** Zeyu Li, Rusli E, Chenjin Lu, Ari Bimo Prakoso, Martin Foldyna, Rasha Khoury, Pavel Bulkin, Junkang Wang, Wanghua Chen, Erik Johnson, Pere i Roca Cabarrocas

**Affiliations:** 1 NOVITAS (Centre of Micro-/Nano-electronics), Nanoelectronics Centre of Excellence, School of Electrical and Electronic Engineering, Nanyang Technological University, 50 Nanyang Avenue, Singapore 639798, Singapore; erusli@e.ntu.edu.sg (R.E.); CLU010@e.ntu.edu.sg (C.L.); ABPRAKOSO@ntu.edu.sg (A.B.P.); 2 LPICM (Laboratory of Physics of Interfaces and Thin Films), CNRS (Centre national de la recherche scientifique), Ecole Polytechnique, Université Paris Saclay, Palaiseau 91128, France; martin.foldyna@polytechnique.edu (M.F.); rasha.khoury@polytechnique.edu (R.K.); pavel.bulkin@polytechnique.edu (P.B.); junkang.wang@polytechnique.edu (J.W.); wanghua.chen@polytechnique.edu (W.C.); erik.johnson@polytechnique.edu (E.J.); pere.roca@polytechnique.edu (P.iR.C.)

**Keywords:** light trapping, silicon thin film, photovoltaics, polystyrene sphere assisted lithography, nanostructured back reflectors

## Abstract

We study light trapping and parasitic losses in hydrogenated amorphous silicon thin film solar cells fabricated by plasma-enhanced chemical vapor deposition on nanostructured back reflectors. The back reflectors are patterned using polystyrene assisted lithography. By using O_2_ plasma etching of the polystyrene spheres, we managed to fabricate hexagonal nanostructured back reflectors. With the help of rigorous modeling, we study the parasitic losses in different back reflectors, non-active layers, and last but not least the light enhancement effect in the silicon absorber layer. Moreover, simulation results have been checked against experimental data. We have demonstrated hexagonal nanostructured amorphous silicon thin film solar cells with a power conversion efficiency of 7.7% and around 34.7% enhancement of the short-circuit current density, compared with planar amorphous silicon thin film solar cells.

## 1. Introduction

By applying plasma-enhanced chemical vapor deposition (PECVD), hydrogenated amorphous/microcrystalline silicon (a/µc-Si:H) tandem solar cells fabricated at low temperatures (around 150 °C) have been researched extensively over the last decade and have achieved over 14% stabilized efficiency [1]. Silicon thin films are promising candidates for future large-scale photovoltaics due to their advantages including lesser material usage, compatible with roll-to-roll processes on flexible substrates, usage of nontoxic and abundant active material, and so on. Effective light trapping mechanisms are of extreme importance in fabricating such high efficient solar cells due to the low absorption coefficient of microcrystalline silicon in the near-infrared region. Nanostructured Ag/ZnO back reflector (BR) substrates are one of the most effective and process compatible approaches for an enhanced light trapping performance in silicon thin film solar cells. A typical BR utilizes metallic or metal/dielectric nanostructures, which produce a far field scattering effect for an extended light traveling path [2,3,4,5], hence a higher absorption. We have previously investigated enhanced light scattering of the BRs patterned by using double size polystyrene (PS) assisted lithography [6]. However, due to plasmonic absorption by the metal/dielectric nanostructures, a significant amount of parasitic losses in the BR is also introduced [7]. To address and characterize such losses, Bruggeman Effective Medium Approximation (BEMA) was previously applied by L. R. Dahal et al. to study the parasitic losses due to a flat or “nanoscale roughness” Ag/ZnO and Al/ZnO interface [8]. However, typical metal/dielectric nanostructures will have dimensions larger than a few tens of nanometers. For example, random nanostructured ASAHI U type substrates with a r.m.s. roughness of around 35 nm are often used in silicon thin film solar cells to enhance light trapping [9]. For random Ag/ZnO BR parasitic losses estimation, extra experimental results are needed to set up an accurate BEMA model. To study the periodic metal/dielectric structures, electromagnetic simulations can be used [10,11]. Not only it is a powerful tool for the design, optimization, and interpretation of the light-nanostructures interactions, but also the absorption enhancement effect in the active layer can be systematically studied and different mechanisms of the electric field enhancement in the system can be readily isolated [12].

In this work, we first systematically studied optical losses in flat Ag/ZnO BRs and random nanostructured Ag/ZnO ASAHI BRs. With different ZnO thickness as the extra variable, we assessed the simulation accuracy of the proposed BEMA model by experimental results. The origin of the parasitic losses in random ASAHI nanostructured Ag/ZnO BRs is concluded. We have used effective medium theory only to describe the effect of random surface roughness of the BRs. After that, by using PS sphere assisted lithography [13], hexagonal nanostructured Ag/ZnO BRs were fabricated. Light scattering properties, as well as optical losses in such BRs were compared with random nanostructured ASAHI BRs. With the help of high frequency electromagnetic field simulator (HFSS), we further investigated the corresponding optical losses in each layer separately. Last but not least, a full device was built upon the BR in the simulation model. Different light scattering modes and each layer’s parasitic losses in the final device were extracted from the simulation results. Photo-generated current was calculated using the AM1.5G solar spectrum. Based on the optical model, we fabricated and characterized silicon thin film solar cells. Experimental results are presented for comparison and verification.

## 2. Experimental Details 

### 2.1. PS Assisted Lithography for Ag Nanostructures on Glass and Hexagonal Back Reflectors

**Figure 1:**
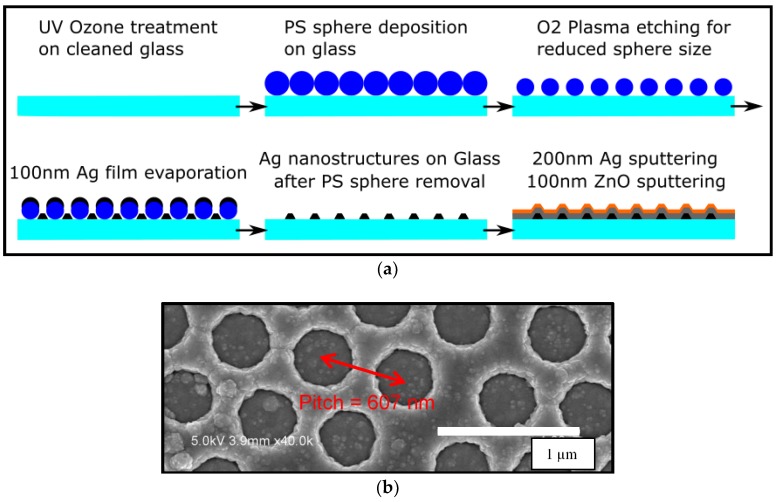
(**a**) Fabrication process and (**b**) Top view SEM image of hexagonal back reflectors (pitch = 607 nm) taken after the final step in Figure 1a.

The PS spheres used in this work were a monodisperse suspension with 5 wt. % in water, obtained from microparticles Gmbh (Berlin, Germany) with nominal diameters of 607 ± 15 nm. We used methanol as the spreading agent, mixed in 1:1 volume ratio with the pristine PS solution. Prior to the PS sphere monolayer transfer, Corning glasses were cleaned with acetone, isopropanol (IPA), and de-ionized (DI) water in an ultrasonic bath, followed by a 10 min UV-Ozone treatment to keep the surface hydrophilic. We used the floating transfer technique for PS spheres monolayer deposition on Corning glass. After that, we used an electron cyclotron resonance (ECR) oxygen plasma etching process for a uniform size reduction of the PS spheres [13]. For the oxygen plasma etching process, we used 40 sccm of O_2_, a pressure of 2.7 × 10^−3^ mbar, and 160 s of etching time at 500 W of plasma power. After thermal evaporation of 100 nm Ag, the substrates were immersed for 20 min in a low power ultrasonic bath in toluene for a complete removal of remaining PS spheres. Another 10 min IPA and DI water ultrasonic bath was applied for a complete removal of any organic solvent residue. Overall fabrication process and SEM image of the hexagonal back reflector are shown in Figure 1. 

### 2.2. Solar Cell Fabrication and Characterization

**Figure 2:**
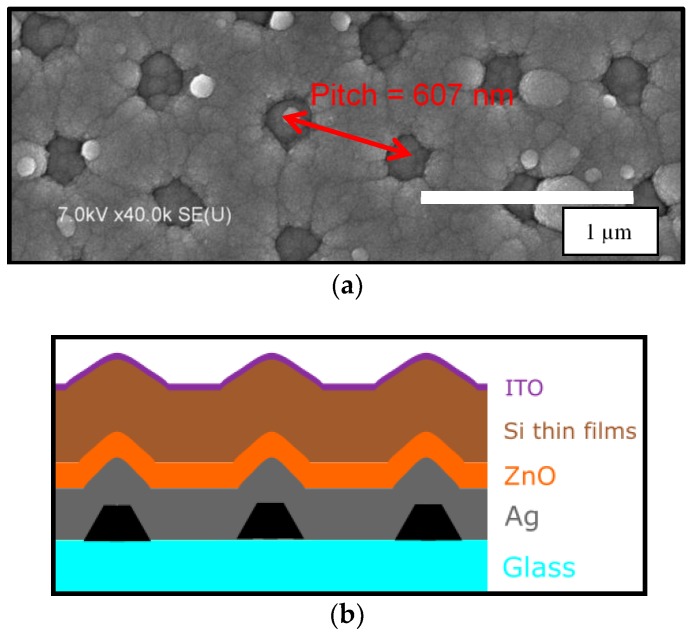
(**a**) Top view SEM image of the complete solar cell and (**b**) Schematic cross section of a hexagonal back reflector solar cell.

Amorphous silicon based p-i-n solar cells were deposited in a radio frequency (RF) PECVD system [14]. First, a 25 nm thick n-type microcrystalline silicon oxide layer was deposited on the BR [15]. Without breaking the vacuum, ~230 nm of intrinsic amorphous silicon layer was deposited using SiH4 plasma at 150 °C, 2 W RF power, and 30 sccm SiH_4_ flow rate, followed by a 25 nm thick p-type amorphous silicon layer. Lastly, 80 nm of indium tin oxide (ITO) was deposited to form the top contacts and to define the cell size of 0.126 cm^2^. Such a fabrication order is often referred to as an n-i-p solar cell [16]. A schematic image of the cross section of the device and its corresponding top view SEM image acquired using a Hitachi S-4700 scanning electron microscope is shown in Figure 2. 

The current density-voltage (J-V) characteristics of the solar cells were measured under AM1.5G illumination with a commercial solar simulator (Oriel AAA, Irvine, CA, USA), calibrated using a crystalline Si reference cell. Total and diffused reflectance were measured using a Perkin-Elmer Lambda 950 spectrophotometer (London, UK) from 350 nm to 1100 nm wavelength range with a 150 mm integrating sphere and an InGaAs detector.

## 3. Results and Discussion

In this section, optical reflectance measurements on a series of flat Ag/ZnO BRs with different ZnO thickness, ASAHI Ag/ZnO BRs, and modeling results based on BEMA will be discussed first. Then, both experimental and HFSS simulation results of the hexagonal Ag/ZnO nanostructured BRs will be presented and compared for absorption mechanism analysis. After that, HFSS simulation results with an active silicon layer on the BR will be presented and analyzed. Last but not least, J-V, external quantum efficiency (EQE), and optical absorption (1 – R_total_ – T_total_) results of the solar cells fabricated on a hexagonal Ag/ZnO nanostructured BR will be compared with the devices made on flat Ag/ZnO BR and on flat ZnO/Corning glass substrates.

### 3.1. Optical Characterization of Flat and ASAHI Back Reflectors

**Figure 3:**
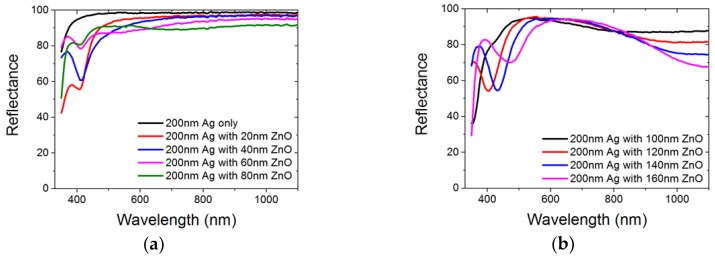
Total reflectance measured on a glass substrate coated with 200 nm Ag and various thicknesses of ZnO (0–160 nm). (**a**) 0–80 nm and (**b**) 100–160 nm.

**Figure 4:**
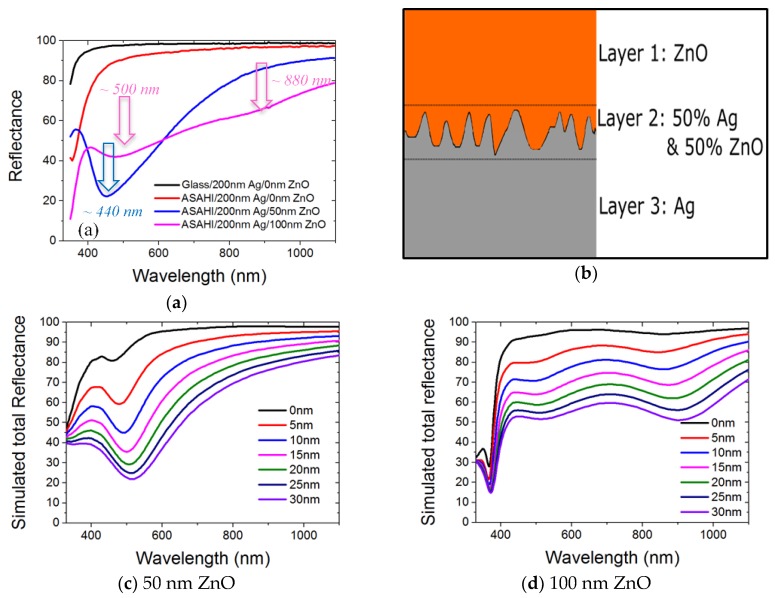
(**a**) Total reflectance measured on ASAHI substrate coated with 200 nm Ag and with 0, 50, and 100 nm of ZnO; (**b**) Bruggeman effective medium approximation model applied for the Ag/ZnO back reflector (BR) study; (**c**) and (**d**) Simulated total reflectance of 50 and 100 nm ZnO (layer 1) with 0 to 30 nm of Ag/ZnO interface (layer 2) thickness.

Before we proceed to study the optical properties of our nanostructured BRs, reference flat Ag/ZnO on glass substrates were fabricated and reflectance data were measured. Figure 3 shows the reflectance data from sputtered ZnO (0 to 160 nm) on 200 nm sputtered Ag on the glass. Figure 4a shows the reflectance data of ASAHI BR with 0, 50, and 100 nm of ZnO. Figure 4b shows the BEMA simulation model we used. Figure 4c,d show the simulated results for various thicknesses of the rough interface layer L2.

#### 3.1.1. Total Reflectance from Flat and ASAHI Back Reflectors

As expected, a pure 200 nm of Ag film on glass provides a highly reflective surface from the 350 nm to 1100 nm wavelength range, with minimum absorption at shorter wavelength. In Figure 3a, we observed that when a thin ZnO was deposited on top of Ag, with its increasing thickness from 20 nm to 100 nm, the local reflectance minimum at 415~420 nm diminishes. D. Sainju et al. [17] concluded that the rough Ag/ZnO interface introduces localized plasmonic absorption at around 2.9 eV due to the nano-protrusions on the Ag surface, which exactly corresponds to local reflectance minimum at 420 nm observed in Figure 3a. With increasing ZnO thickness from 20 nm to 80 nm, such a local reflectance minimum becomes less significant. A previous study has reported that refractive index of sputtered ZnO thin film are different when deposited on different substrates [18]. E. Moulin et al. concluded that for thin film silicon solar cells with a Ag BR structure, dielectric material with a lower refractive index between Ag and active silicon thin films results in lower optical losses in BRs [19]. From [20], the author argues that when an interference minimum in reflectance matches the plasmon energy of the rough Ag/ZnO interface, this will lead to a strong coupling of the energy from the optical field into the plasmon mode. With the increased film thickness, the interference minimum shifted away from 420 nm due to Fabry-Perot resonance. Such a shift has led to a decreased plasmon absorption, hence a decreased local reflectance minimum at 420 nm is observed.

The shifts of the interference minimum due to Fabry-Perot resonance are easier to observe in Figure 3b. From Figure 3b, when a thicker ZnO was deposited on top of Ag, with the increasing thickness from 120 nm to 140 nm to 160 nm, reflectance minimum shifts from 400 nm to 430 nm to 470 nm. By using the refractive index of crystalline ZnO on metal from reference [18], we can calculate the condition of destructive interference using

λ_destructive = (2 × *n*_ZnO × *t*_ZnO × cos (7°))/(*m* − 1/2) (1)

where *m* = 1 for the first and *m* = 2 for the second destructive interference respectively. At 120 nm ZnO thickness, by using n_ZnO = 2.46 at 400 nm wavelength [18] (where we suspect 400 nm is the 2nd destructive inference), the calculated λ_2nd_destructive at *m* = 2 is 390 nm. This calculation matches the observation of the red shifting of the reflectance minima with increasing ZnO thickness. The difference between the calculated reflectance minima and our experimental data might be due to the difference in the ZnO refractive index when deposited on different substrates; the metal substrate used in reference 14 is Pt, while we are using Ag in our case. The first destructive interference wavelength λ_1st_destructive is calculated to be 1010 nm, by using n_ZnO = 2.12 at 900 nm wavelength [18], *m* = 1. This further explains the reduced reflectance for the wavelength range from 900 nm and beyond in Figure 3b. Other than above, the increased free carrier absorption of a thicker ZnO layer will also play a role in the overall optical performance [21]. From the experimental data, to avoid parasitic absorption by the BRs in the longer wavelength range, 100 nm of ZnO might be more desirable when compared with thicker ZnO cases, as the thickness of ZnO determines the absorption behavior of flat Ag/ZnO BRs. Special care shall be taken at ZnO thickness when BRs are nanostructured.

With the above understanding of flat Ag/ZnO BRs performance, the reflectance results from ASAHI BRs are presented in the next section. In Figure 4a, 200 nm of Ag sputtered on rough ASAHI substrate shows a lower reflectance compared with 200 nm of Ag sputtered on flat glass substrate. This is due to a higher plasmonic absorption on a rough Ag surface [22]. With 50 nm of sputtered ZnO on top, significant absorption is observed for all wavelengths with local reflectance minimum at around 440 nm. With 100 nm of sputtered ZnO on top, reflectance further reduces from the 600 to 1100 nm wavelength range but partially recovers from the 400 to 600 nm range. Two local minima at around 500 and 880 nm have been observed. Such large differences in optical performance between flat BR and ASAHI BR shall be fully understood before we proceed to study the more complex hexagonal nanostructured Ag/ZnO BRs. Other than the total reflectance properties, the effect of nanostructure morphology on diffused reflectance will also be studied and presented in later sections.

#### 3.1.2. Bruggeman Effective Medium Approximation Simulation

In this section we use the Bruggeman Effective Medium Approximation (BEMA) method to estimate the optical performance of the random nanostructured ASAHI Ag/ZnO BRs. BEMA is a fast simulation method for the macroscopic properties of composite materials, which we use to simulate Ag and ZnO mixture in BRs. With a 35 nm r.m.s. roughness on ASAHI substrate surface [9], one would expect less than 35 nm surface roughness at Ag/ZnO interface with added smoothing effect of the sputtered Ag layer [23]. To estimate the Ag/ZnO interface roughness and to correlate between interface roughness and the optical performance, BEMA simulation is used to study the random nanostructure ASAHI Ag/ZnO BRs. Figure 4b shows the BEMA simulation model we used. We designed our simulation model such that Ag/ZnO interface roughness is modelled as a thin film layer (L2) composed of 50% Ag and 50% ZnO, in between the pure ZnO layer (L1) and pure 200nm Ag layer (L3). By assuming a uniform Ag surface roughness and also a conformal coating of the ZnO on top, 50% to 50% composition ratio of the two materials and 0% void should be a good approximation to model the interface roughness layer. Figure 4c,d show the simulated reflectance with 50 and 100 nm of ZnO layer respectively. Interface roughness, that is, the thickness of L2, was changed from 0 to 30 nm in steps of 5 nm.

With 50 nm of ZnO, comparing between the experimental results in Figure 4a and simulation results in Figure 4c, we can see that in terms of the absolute magnitude (~20%), the reflectance from ASAHI BRs is close to the case of simulated 25 nm interface roughness. However, in terms of the position of the local reflectance minima, the reflectance from ASAHI BRs is close to the case of simulated 10 nm interface roughness. Similarly, in the case of 100 nm ZnO, a simulated 25 nm interface roughness shows closer results in absolute magnitude, and a simulated 10 nm interface roughness shows a closer result to the experimental data in position of the local reflectance minima. The above observations can be explained considering that with increasing Ag/ZnO interface layer thicknesses, the effective overall ZnO thickness will be increased. The red shift of the reflectance minima is due to the increased ZnO thickness.

From the simulation results above, we can observe that ZnO thickness (L1) determines the shape of the reflectance curve, while Ag/ZnO interface thickness (L2) determines the magnitude of the reflectance value. We suggest that top ZnO (L1) governs the interference behavior of the incident light. Ag/ZnO interface (L2) on one hand could absorb light which reduces the overall reflectance, and on another hand could be effectively treated as an antireflection layer in between ZnO and bulk Ag. Such an antireflection effect causes more light to be transmitted into bulk Ag (L3), hence more parasitic absorption. From Figure 4c,d, with a uniform increasing L2 thickness, we observe a uniform reduction in reflectance for the wavelength range around 1000 nm. However, in the wavelength range around 500 nm, the reduction of reflectance is large when L2 is thin (15% reduction from 5 to 10 nm) but small when L2 is thick (5% reduction from 25 to 30 nm). This can be explained by the antireflection effect, which saturates with increasing L2 thickness. The saturation in absorption loss of the BRs has been reported by J. Springer et al. [24], while we propose a new possibility to explain such behavior using BEMA approximation.

Our model predicts that such BR has an interface thickness of 25 nm. Surprisingly if we compare the simulated 5 nm interface roughness (100 nm ZnO layer 1) with 100 nm ZnO on 200 nm Ag reflectance data in Figure 3, we can also observe a similar reflectance magnitude and local minima position. Hence, from the perspective of our simulation results, the nominally flat Ag/ZnO BR also has a 5 nm Ag/ZnO interface thickness. The ZnO/air surface roughness has been investigated as well, but no significant changes in the reflectance behavior were observed.

### 3.2. Experiment and Simulation Results on Hexagonal Ag/ZnO Nanostructured Back Reflectors

**Figure 5:**
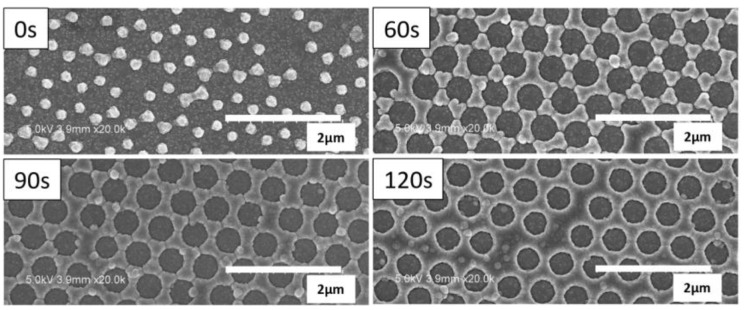
SEM images of hexagonal Ag/ZnO back reflectors obtained with 0, 60, 90, and 120 s of O_2_ plasma treatment of polystyrene spheres (See Figure 1a for process flow).

In previous sections, we have presented the study on the flat and random nanostructured ASAHI BRs. Their optical loss mechanisms were investigated and correlated with the thickness of ZnO layer as well as Ag/ZnO interface layer. In this part, hexagonal nanostructured BRs are studied. Figure 5 shows the top view of the BRs with different O_2_ plasma etching times (see Figure 1a for the process flow).

BEMA is not appropriate to describe the optical behavior of our hexagonal nanostructured BRs. For such BRs, with a semi-periodic arrangement of the metal/dielectric nanostructures and close-to-visible wavelength inter-nanostructure distance, we expect more plasmonic modes to be present in the system, hence higher optical losses in the hexagonal nanostructured BRs compared with random ASAHI BRs. However, a higher optical loss of the nanostructured BR might not necessarily mean a lower quality. For the nanostructured BRs, light scattering capability is another figure of merit that might be more crucial for one to take into consideration. The diffused reflectance is an indirect way to characterize the scattering capability of the BRs [25]. To further investigate, not only the total reflectance but also the diffused reflectance data were collected. 

#### 3.2.1. Total and Diffused Reflectance of Hexagonal Ag/ZnO Nanostructured Back Reflectors

**Figure 6:**
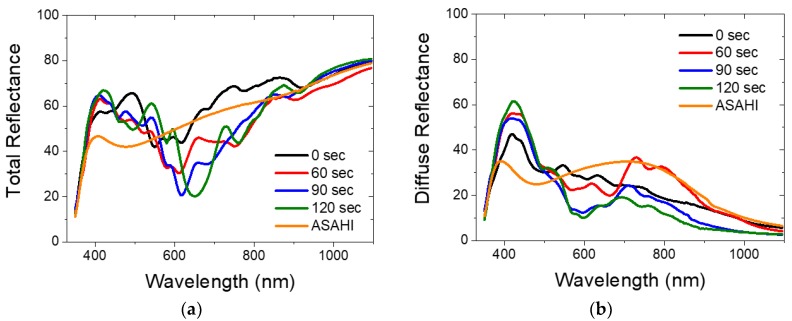
(**a**) Total and (**b**) diffused reflectance of hexagonal Ag/ZnO BRs and ASAHI BR with 100 nm ZnO.

Figure 6a,b show the total and diffused reflectance of the hexagonal nanostructured BRs and ASAHI BR with 100 nm ZnO (see Figure 5 for SEM images). Similar optical performance of the different BRs is observed. Comparing with ASAHI BR, we observe higher total reflectance as well as higher diffuse reflectance from around 370 nm to around 570 nm range. This shows that for the above range, hexagonal nanostructured BRs not only have a lower optical loss but also have a stronger light scattering capability. However, from around 570 nm to 840 nm range, which is more critical for light trapping in silicon thin film solar cells, the total reflectance of hexagonal nanostructured BRs is significantly lower than that of the ASAHI BR, especially for the BRs fabricated using O_2_ plasma etching. This indicates that, other than Ag/ZnO interface roughness, additional absorptions are present in the hexagonal nanostructured BRs. Due to such significant additional absorptions, the amount of light that is diffused is reduced as well. Hence, for around 570 nm to 700 nm, the diffuse reflectance values of hexagonal nanostructured BRs are lower than ASAHI BR. For the 60 s hexagonal nanostructured BR case, from 700 to 900 nm range, a similar or even higher diffuse reflectance compared to ASAHI BR is obtained (Figure 6b), regardless of the observed higher optical losses on the 60 s hexagonal nanostructured BR. What’s more, with the increasing etching time, we notice a decrease in the diffused reflectance, and a red shift in the total reflectance. To understand the source of the reduced reflectance at different local minimum and the effect of the etching time for the losses in total reflectance, the HFSS model was designed and simulation results were extracted and compared with the experiment results.

#### 3.2.2. Simulated Absorption of Hexagonal Ag/ZnO Nanostructured Back Reflectors

**Figure 7:**
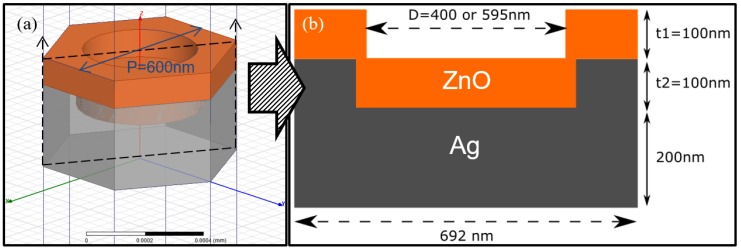
Simulated hexagonal nanostructured back reflector; (**a**) HFSS unit cell in the simulation model and (**b**) corresponding cross sectional view.

Figure 7a,b shows the unit cell in the simulation model and corresponding cross sectional view of the BRs we designed in HFSS. Due to the periodic boundary conditions placed onto the six sides of the unit cell, an infinite large area is simulated by repeating the unit cells next to each other. Such hexagonal nanostructure has a fixed periodicity of 600 nm (P), which is the diameter of the PS spheres we used during fabrication. The opening of the nanostructures (*D*) is set to be either 595 nm or 400 nm. At *D* = 595 nm, the opening of the nanostructure is close to the initial diameter of the PS sphere. We use such a condition for the approximation of 0 s etched BR performance estimation. For the 120 s etched BR case, the opening diameter is measured from the SEM image and approximately to be 400 nm. Hence *D* = 400 nm is used for approximation of the 120 s etched BR performance estimation. The ZnO thickness *t*_1_ was set at 100 nm, below Ag hexagonal nanostructures thickness *t*_2_ was set at 100 nm. Bottom bulk Ag thickness was set at 200 nm.

**Figure 8:**
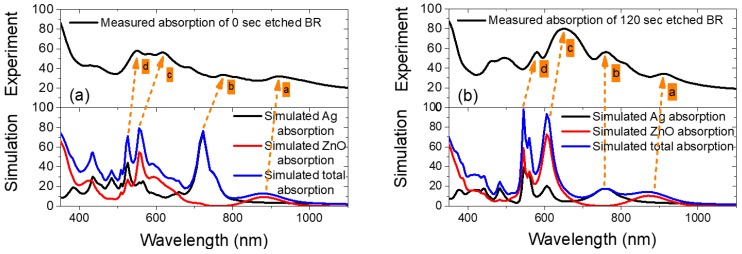
(**a**) 0 s etched BR measured absorption (top) compared with a simulated absorption under 595 nm opening (*D* = 595 nm) simulation case; (**b**) 120 s etched BR measured absorption (top) compared with simulated absorption under 400 nm opening (*D* = 400 nm) simulation case.

Figure 8 shows the comparison of the measured absorptance (1 *– R*) for 0 and 120 s etched BRs and simulated absorption in each layer for 595 and 400 nm opening models. Other than the absorption by ZnO at its optical band gap around 360 nm, four distinctive absorption peaks (a–d highlighted in yellow) are observed both from experiment and simulation results. The peak marked a in Figure 8 is attributed to the ZnO bulk absorption [18]. The peak b is attributed to the Ag plasmonic resonance absorption, which shifts with the opening size. However, this peak did not contribute to overall BR absorption significantly in the experiment. This might be due to the fact that in real devices, the semi-periodic nanostructures did not produce a strong plasmonic effect as compared with the simulation case where they are rigorously periodic. Peaks c and d near 500–600 nm are mainly due to ZnO absorption which was enhanced by the nanostructured Ag below [26]. A consistent red shift of the experimental results with respect to the simulations is observed. This might be due to the minor difference in ZnO film thickness between experiment and simulation model.

With the help of HFSS simulation, we managed to assign each of the optical losses in the periodical hexagonal nanostructured Ag/ZnO BR. Other than the rough Ag/ZnO interface absorption as modelled by the BEMA method, ZnO absorption enhanced by the bottom Ag nanostructure also contributes significantly to the BR absorption. The significant background absorption in NIR region for the experimental results, which differs by about 20% simulation, are attributed to Ag plasmonic absorption due to rough surfaces.

### 3.3. Experiment and Simulation Results on Hexagonal Ag/ZnO Back Reflector Solar Cells

**Figure 9:**
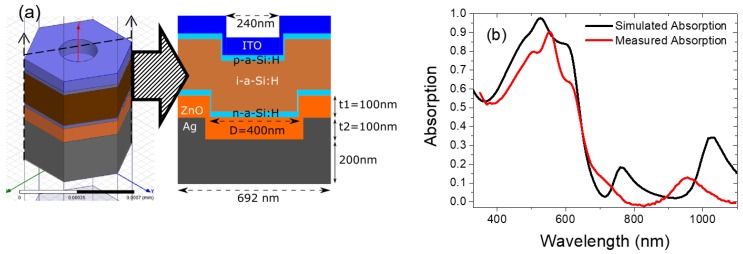
(**a**) High frequency electromagnetic field simulator (HFSS) simulation model for hexagonal BR solar cell; (**b**) Simulated and measured absorption of the plannar Ag/ZnO BR solar cell.

To investigate the potential parasitic losses from the BRs in solar cell devices as well as to understand different light scattering modes that appeared due to such nanostructures, 25 nm n-type amorphous silicon, 230 nm intrinsic amorphous silicon, 25 nm p-type amorphous silicon, as well as 80 nm top ITO are added on top of the 400 nm opening BR. The 400 nm opening of the BR and final 240 nm opening of the finished device is measured from SEM images in Figure 1b and Figure 2a respectively. This model simulates the 120 s O_2_ plasma etched BR and its corresponding solar cell. Due to additional layers added in the model, parasitic losses will be introduced not only by the BRs, but also from ITO and the recombination in highly n/p doped layers. Indeed, those layers will also absorb light but will not contribute to photo-current generation. Figure 9a shows the unit cell and the corresponding cross section of the simulation model of a hexagonal BR solar cell. The overall absorption and individual layers’ absorption are simulated.

To validate our simulation results, simulated overall absorptance, and measured absorptance (1–measured reflectance) of the flat Ag/ZnO BR solar cell device are plotted in Figure 9b. We can observe similar absorption trend between simulation and experiment. Again experimental results are slightly red shifted, due to the minor difference in film thickness (including ITO, ZnO, and silicon thin films) between experiment and simulation model. Additional contribution to increased absorptance in measured data might be reduced reflectance due to surface roughness, which were not been included in the simulation. In the next section, we present the simulation results of hexagonal nanostructured Ag/ZnO BR solar cells.

#### 3.3.1. Parasitic Losses and Absorption Enhancement Analysis

**Figure 10:**
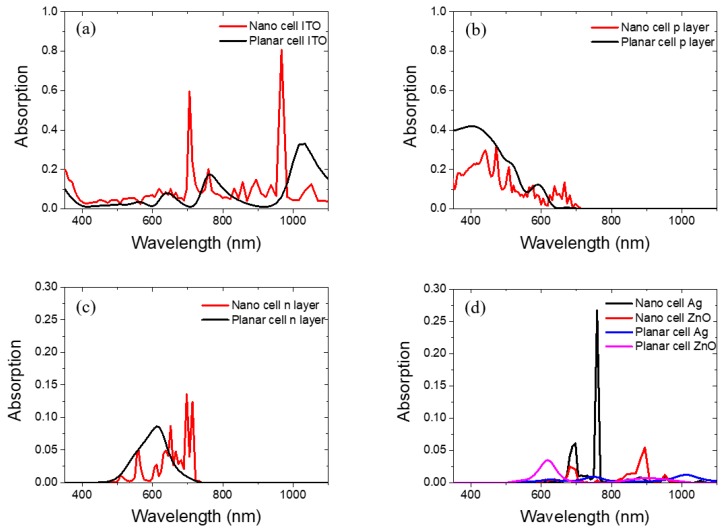
(**a**) Indium tin oxide (ITO) absorption; (**b**) p layer absorption; (**c**) n layer absorption; (**d**) Ag/ZnO BR absorption.

To analyze the parasitic losses in the different layers of the n-i-p solar cell and the absorption enhancement in the intrinsic silicon layer, the base line for us to compare is the simulated planar device. Based on the difference between planar and nanostructured solar cells, solid conclusions on the parasitic losses, absorption enhancement, and light scattering modes can be drawn. Figure 10a–d shows the simulated ITO, p-type silicon, n-type silicon, and BR absorption for both planar and nanostructured solar cells. From the results we can see that after the solar cells are nanostructured, ITO absorption is greatly enhanced, p layer absorption is reduced, and n layer absorption is reduced from 500 to 650 nm but enhanced from 650 to 720 nm range. For the Ag and ZnO layers, our results show that in planar BR solar cell, Ag almost does not absorb any light and minimum absorption around 620 nm is observed for ZnO. For the nanostructured BR solar cell, ZnO absorption is reduced and red shifted to 690 nm. Two peaks at 700 and 760 nm are observed for Ag. These two absorption peaks will contribute to parasitic absorption as well. Unlike the above results where high absorption is observed for the BRs in air medium, when silicon active layers are deposited on top, BR parasitic absorption is less important as compared to the top ITO parasitic absorption.

**Figure 11:**
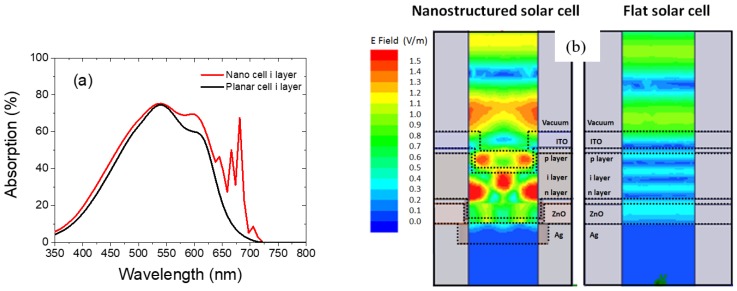
(**a**) Simulated intrinsic amorphous silicon absorption; (**b**) E field distribution of nanostructured and planar solar cell at 670 nm.

To study the absorption enhancement effect, intrinsic amorphous silicon absorption is plotted in Figure 11a for planar and nanostructured cases. We can observe that the absorption in the intrinsic layer for the nanostructured solar cell is significantly enhanced not only in the short wavelength range (350–520 nm) but also for longer wavelength range (550–720 nm). With referring to the formula below [27] and by using 1.71 eV band gap for amorphous silicon and the AM1.5G solar spectrum, we calculated the generated photo-current density to be 18.3 mA/cm^2^ and 15.0 mA/cm^2^ for a nanostructured and planar solar cell respectively. In the formula below, *I*(λ) is the spectral irradiance of the standard AM1.5G solar spectrum, *A*(λ) is the absorption in the active absorber layer, and *E*(λ) is the energy of the photon at its corresponding wavelength:

*J*_SC_ = ∫_300 nm to 1200 nm_ {[E_g_ × I(λ) × A(λ)]/E(λ)} dλ (2)

To characterize the light resonance mode for the nanostructured solar cell, we selected 670 nm where a sharp absorption enhancement peak is observed in Figure 11a and we compared the electric field distribution in the nanostructured and planar solar cell. Figure 11b shows the electric field distribution at 670 nm incident wavelength. From Figure 11b, we can clearly see the light trapping effect of the nanostructured solar cell. Multiple “hot spots” are induced. The generation of those “hot spots”, that is, high electric field strength regions, is due to interferences and waveguide modes. Strong resonance modes are induced inside intrinsic layer. Hence the absorption in the intrinsic layer is strongly enhanced.

#### 3.3.2. Experimental Results

**Figure 12:**
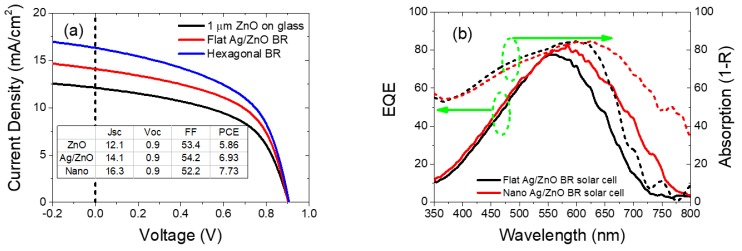
(**a**) Density-voltage (*J*-*V*) and solar cell parameters of different solar cells; (**b**) external quantum efficiency (EQE) and absorption (1 – R_total) of nanostructured and planar Ag/ZnO BR solar cells.

Last but not least, we fabricated solar cells using conventional 1 µm ZnO on glass substrates, Ag/ZnO flat BR substrates, and hexagonal nanostructured Ag/ZnO BR substrates. The experimental results are compared and analyzed. Figure 12a shows the *J*-*V* characteristics and a table summarizing their parameters. Figure 12b shows the EQE and optical absorption data (1–total reflectance) of the planar Ag/ZnO BR and hexagonal nanostructured Ag/ZnO BR solar cell.

From Figure 12a, we observe an increasing short-circuit current density. With the incorporation of flat Ag/ZnO BR, 2.0 mA/cm^2^ of short-circuit current density are gained compared to the transparent ZnO on a glass substrate. The current collection is enhanced by 16.5%. With the incorporation of hexagonally nanostructured Ag/ZnO BR, not only is the open-circuit voltage not compromised but also the short-circuit current density is further enhanced by 2.2 mA/cm^2^. The total Jsc enhancement compared to the reference transparent flat ZnO substrate is as high as 34.7%. From EQE and absorption results in Figure 12b, we observed the enhanced light absorption from 550 to 750 nm wavelength range. This indicates an improved light collection efficiency by incorporating the nanostructured BR into the flat BR device, which leads to an enhanced short-circuit current. Notably, for the flat Ag/ZnO BR substrate we find good agreement between the measured short-circuit current density in the experiment and the calculated short-circuit current by simulation (6% difference). However, simulation predicted an 18.3 mA/cm^2^ short-circuit current density for the hexagonal nanostructured solar cell, while experimentally we only obtained 16.3 mA/cm^2^ (10.9% difference). Such difference is expected, as in our ideal simulation model, electrical resistivity, defects at the n/i interface, i/p interface, as well as bulk defects in the intrinsic layer are not considered and the collection efficiency of charge carriers is assumed to be 100%. Furthermore, the nanostructured solar cells have a larger contact area compared to the planar solar cells. Hence we would expect more interface defects, which leads to a larger simulation to experiment difference for the nanostructured solar cell than the flat solar cell. From Figure 12b, for the EQE of our real devices, resonance modes are broadened due to the semi-periodic arrangement of the nanostructures and a smooth and continuous light trapping effect from 550 to 750 nm is observed. While in simulated intrinsic silicon absorption, strong resonances at specific wavelengths contribute significantly to the collected current. This is due to the periodicity of the feature being rigorously achieved during the calculation, hence strong light trapping effects are observable only at specific wavelengths. Paetzold et al. [28] reported a series of systematic study and proved that disordered periodic are superior than the rigorous periodic nanostructured BRs for silicon thin film solar cell. While in our study, the reasons why the experimental results are not better than the simulation results are mainly due to the imperfect and unrealistic simulation model which excluded electrical losses. Even though we cannot conclude that our semi periodic nanostructured BRs are better than the rigorous periodic nanostructured BRs, nevertheless, the method used in this work is a good demonstration to achieve a quasi-periodic nanostructures using cost effective PS sphere assisted lithography. The distorter that contribute to the quasi-periodic nanostructures in our work is originated from the deviation of close-packed hexagonal assembly of PS spheres, as well as deviation from oxygen plasma etching of the PS materials.

## 4. Conclusions

In this work, we have demonstrated a novel hexagonal nanostructured design and fabrication process for light trapping in silicon thin film solar cells. The hexagonal nanostructured back reflectors and corresponding solar cells were modeled by the finite element method and verified against experimental data. The parasitic losses in the back reflector mainly come from the rough Ag/ZnO interface plasmonic absorption and enhanced ZnO absorption by Ag nanostructures. The high frequency electromagnetic field simulator was proven to be an effective tool for modeling our hexagonal nanostructured back reflectors and solar cells. Simulation results show similar trends with experimental data. From simulation, parasitic losses in our nanostructured solar cells mainly come from the ITO top contact layer, while back reflector parasitic losses are less significant. Calculated photo generated current values are close to the experimental short-circuit current, with 6.0% and 10.9% differences for the planar and nanostructured solar cells respectively. Such a difference is attributed to certain parameters that were not considered in the simulation, such as electrical resistivity, defects in the bulk and interfaces. Furthermore, our fabrication approach is not limited to a-Si:H silicon thin film solar cells, but can also be applied to µc-Si:H silicon thin film solar cells. As a low cost, robust, and process compatible method, these nanostructured BR strategies are an effective candidate for improved light trapping performance in silicon thin film solar cells.
